# Trickledown Effect?: Maternal Alcohol Consumption Linked to Cryptorchidism in Sons

**Published:** 2007-02

**Authors:** Julia R. Barrett

Cryptorchidism (undescended testes), the most frequently occurring genital malformation in newborn boys, is a risk factor for later testicular cancer and fertility problems. By some reports, incidence has increased in recent decades, with environmental and lifestyle factors as potential contributors. As part of a broader investigation of these factors, a study of alcohol consumption during pregnancy reveals that imbibing five or more drinks per week may increase the risk of cryptorchidism **[*EHP* 115:272–277; Damgaard et al.]**.

Prenatal alcohol exposure has already been linked to low birth weight and fetal alcohol syndrome, a spectrum of neurological and developmental problems. It may also be associated with abnormalities of the bones, heart, and genitourinary tract. Health experts generally advise women to avoid alcohol in pregnancy because research has not identified a safe level of consumption. Defining health effects due solely to alcohol consumption is complicated, though, owing to numerous confounding factors. For example, mothers in the current study who drank alcohol were also more likely to smoke, a factor associated with low birth weight, which in turn is linked to cryptorchidism.

The researchers used prospectively collected medical history and lifestyle data from 4,957 pregnant women in Denmark and Finland. These women had completed a self-administered questionnaire by the beginning of the third trimester of pregnancy, answering questions on alcohol, coffee, and tea consumption, as well as smoking. The 2,475 participating women gave birth to 2,496 sons, who were examined for cryptorchidism at birth and at three months. At birth, 128 boys had varying degrees of cryptorchidism, and at three months 33 remained cryptorchid.

Half the boys with transient cryptorchidism and nearly 70% of those with persistent cryptorchidism were born to mothers who did not drink at all. Nevertheless, data analysis showed that mothers who regularly drank alcoholic beverages during pregnancy were more likely to have sons with cryptorchidism, with an apparent dose–response trend. The association held even after controlling for smoking, maternal age, birth weight, caffeine intake, and other potential confounders.

Although adverse effects were not statistically significant below five drinks per week, the researchers caution that their data do not support any conclusion regarding a safe level of drinking during pregnancy. They also cannot rule out some contribution to overall adverse effects from examined confounders, such as smoking, as well as those for which data were not collected, such as diet.

## Figures and Tables

**Figure:**
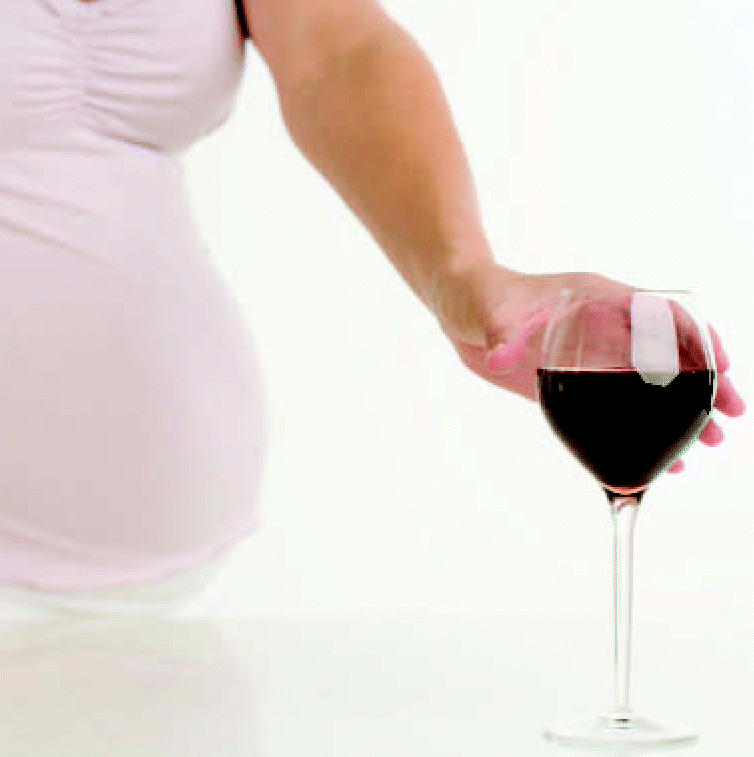
Booze and boys A new study links alcohol consumption during pregnancy with reproductive effects in sons.

